# Cyberbullying in the University Setting. Relationship With Emotional Problems and Adaptation to the University

**DOI:** 10.3389/fpsyg.2019.03074

**Published:** 2020-01-21

**Authors:** María Carmen Martínez-Monteagudo, Beatriz Delgado, José Manuel García-Fernández, Cecilia Ruíz-Esteban

**Affiliations:** ^1^Department of Developmental Psychology and Didactic, Faculty of Education, University of Alicante, Alicante, Spain; ^2^Department of Developmental and Educational Psychology, Faculty of Psychology, University of Murcia, Murcia, Spain

**Keywords:** cyberbullying, anxiety, depression, stress, adaptation to university

## Abstract

Little scientific attention has been paid to the problem of cyberbullying in the university environment, compared to similar studies conducted on adolescents. This study attempts to analyze the predictive capacity of certain emotional problems (anxiety, depression, and stress) and university adaptation with respect to cyberbullying in victims and aggressors. The European Cyberbullying Intervention Project Questionnaire, the Depression Anxiety Stress Scale-21 and the Student Adaptation to College Questionnaire were administered to a sample of 1282 university students (46.33% male) aged between 18 and 46. The results suggest that high levels of depression and stress increase the probability of being a cyberbullying victim, while high levels of depression increase the probability of being a cyberbullying aggressor. Similarly, the personal–emotional and social adaptation of students are found to be predictor variables of being a cyberbullying victim, in that high levels of personal–emotional and social adaptation decrease the probability of being a victim, while high levels of personal–emotional, academic and institutional adaptation decrease the probability of being a cyberbullying victim. The results of this study are of special relevance, since they indicate that intervention programs should consider the influence of emotional intelligence, as well as the relevance students’ adaptation to university.

## Introduction

Currently, an increasing number of studies are examining the issue of cyberbullying in the university environment (Faucher et al., 2014; Zalaquett and Chatters, 2014), revealing that higher education is not free from this phenomenon. Cyberbullying is defined as “willful and repeated harm inflicted through the use of computers, cell phones, and other electronic devices” (Hinduja and Patchin, 2009, p. 5). The widespread use of information and communication technologies, especially by young people, has transformed the way that society accesses information and therefore, how we communicate and interact with others. Clearly, there are benefits to this; however, there are also newly arising problems associated with the inappropriate use of these new technologies. Thus, some of the phenomena that are commonly associated with the school environment such as bullying have found their equivalents in new virtual scenarios or realities. Cyberbullies, who are highly skilled in these new digital scenarios, rely on these technologies to carry out aggressive behavior toward their peers (threats, harassment, bribes, insults, humiliation, publication of confidential information, identity theft, manipulation of photographs, recording, and dissemination of physical aggressions, etc.).

Many studies have analyzed the phenomenon in adolescence (DeSmet et al., 2015; Ho et al., 2017; Extremera et al., 2018; Quintana et al., 2019), however, lately, high prevalence rates have also been found in the university environment. So, researchers have suggested that prevalence rates of students who are victimized by electronic means during the higher education period may range from 5 to 40% (Finn, 2004; Lindsay and Krysik, 2012; Faucher et al., 2014; Zalaquett and Chatters, 2014). Finn (2004) using a broad sample of 2002 United States university students found that between 10 and 15% reported having received threatening emails or messages, with insults or harassment. Lindsay and Krysik (2012) found higher prevalence rates in a sample of 420 university students, suggesting that 43.3% indicated having suffered from cyberbullying. Faucher et al. (2014) using a sample of 1925 Canadian university students, found that 24.1% reported having been a victim of cyberbullying over the past year. Zalaquett and Chatters (2014) found that 19% of a sample of 613 university students reported having been victims of cyberbullying, whereas 5% reported having been cyberbullying aggressors. The variability in the prevalence rates is quite high, perhaps due to the different conceptualizations of cyberbullying, the distinct methodologies used or the determination of the frequency that is necessary in order to be considered cyberbullying. Despite this variability, empirical evidence confirms the presence of this problem in the university setting.

So, cyberbullying has become a concerning social issue, given its high prevalence and serious repercussions (Kowalski et al., 2012), leading to research intended to identify its potential predictors in an attempt to prevent and intervene in this area. Generally speaking, cyberbullying results from the interaction between the student’s personal characteristics and the development contexts in which these characteristics unfold (Benbenishty and Astor, 2005). The university context and the changing life phase of the students during this period have some specific characteristics that differ from other life phases (childhood or adolescence) or educational cycles (primary or secondary education) which may influence cyberbullying and should therefore, be closely considered. The majority of university students are undergoing a new and changing life phase that is referred to as emerging adulthood (Arnett, 2008) in which new behavioral, cognitive and social or affective responses are being developed in response to new environmental demands. The university phase coincides with this new adult role, as it demands the handling of new scenarios that are marked by the separation from parents and friends, the creation of new social circles and the need for increased autonomy and responsibility. So, along with increased academic demands that may mark their future in the labor world, university students also face additional social and emotional challenges. Against this backdrop, many studies have found that a high number of university students face academic or emotional problems (American College Health Association, 2013). Depression, anxiety and stress have been identified in a high percentage of this population (Arrieta et al., 2013; Beiter et al., 2015; Dalky and Gharaibeh, 2018). Arrieta et al. (2013) found that the prevalence of symptoms of anxiety, depression and stress in Colombian university students was 37.4%, 56.6%, and 45.4%, respectively. Beiter et al. (2015), using a sample of 374 United States university students, found that 11%, 15%, and 11% presented severe or extremely severe levels of stress, anxiety and depression, respectively. Dalky and Gharaibeh (2018) reported even higher prevalence rates in their sample of 600 university students. Of these students, 43.2%, 58.2%, and 25.3% displayed severe or extremely severe levels of depression, anxiety and stress, respectively. Psycho-social factors and characteristics of university life (competitive academic environment, excess work, lack of solid peer relationships, pending unemployment, etc.), which are significantly distinct from those of other education levels, are associated with a decrease in student mental health (Misra and McKean, 2000; Kumaraswamy, 2013). On the other hand, regarding the relationship between cyberbullying and emotional problems (anxiety, depression and stress), a considerable number of empirical studies have found high rates of these problems in cyberbullying victims. So, the majority of studies have suggested that these students display high levels of anxiety, depression, stress, low self-concept, powerlessness, somatization, loneliness, anger, sleep disorders, concentration problems low academic performance, and absenteeism (Schenk and Fremouw, 2012; Faucher et al., 2014; Giménez et al., 2015; Na et al., 2015; Aricak and Ozbay, 2016), and even suicidal ideation (Hinduja and Patchin, 2010; Schenk and Fremouw, 2012; Jasso et al., 2018) as a result of being a victim of bullying, humiliation, harassment, etc., while the bullies display externalizing behaviors, low empathy, aggressive behavior, drug abuse and truancy (Hinduja and Patchin, 2007; Aricak, 2009), as well as anxiety, depression, psychosomatic symptoms, and suicide (Nansel et al., 2001, 2003; Seals and Young, 2003). Thus, in the university setting, emotional problems may be found in a high percentage of cases, without necessarily being associated with cyberbullying, although being a cyberbullying victim or aggressor may lead to the development of high levels of anxiety, depression and stress in students, establishing complex relations between these constructs. So, while some students experience high levels of anxiety, depression and stress during this specific academic period of change which makes them more vulnerable to suffering from or engaging in acts of cyberbullying, many of these emotional problems may result from being a victim or aggressor of cyberbullying. This latter assumption has been widely corroborated in the scientific literature (Schenk and Fremouw, 2012; Faucher et al., 2014; Aricak and Ozbay, 2016), especially with respect to the victims. So, anxiety, depression, stress and other emotional problems may be the result of the victimization, but it is also possible that depressed students, having a high level or anxiety or stress, may become victims of intimidation given their inappropriate social behavior, lack of self-esteem or inability to defend themselves due to the depression, anxiety or stress that they are experiencing. Similarly, a depressed student with high levels of anxiety or stress may have a low level of peer acceptance, leading to externalizing behaviors, and thereby turning them into aggressors. So, many studies have shown that the association between school bullying and internalizing and social problems is reciprocal (Hodges and Perry, 1999; Sweeting et al., 2006); however, there is inconsistency with respect to the direction of causality (Kaltiala-Heino et al., 2010). To conclude, although it has been widely demonstrated that suffering from cyberbullying or being a cyberbully may result in emotional problems, there is limited empirical evidence regarding the relationship between suffering from emotional problems and its predictive capacity for being a cyberbullying victim or aggressor. Similarly, most of these studies have focused on adolescent populations, with few studies considering the university population.

On the other hand, in addition to the student’s personal variables, some studies note contextual variables such as those involved in student adaptation to the academic environment. Thus, although adjustment to the university setting has been conceptualized from distinct perspectives, one of the most widely accepted perspectives was established by Baker and Siryk (1984, 1999) and Baker et al. (1985), revealing that university students tend to experience distinct types of adjustment to the university: academic adaptation (fulfilling the university’s educational demands and obtaining good results), social adaptation (confronting the interpersonal demands of the university), personal–emotional adaptation (feeling good physically and psychologically), and the institutional link (feeling good about the university in general and having a quality bond with the selected institution). Many studies have suggested that poor adjustment to the university may have considerably negative effects on the student (Friedlander et al., 2007; Credé and Niehorster, 2012). In a meta-analysis, Credé and Niehorster (2012) found that students who do not adapt well to the university present poorer academic performance, fewer probabilities of completing their studies and a greater tendency to seek counseling services and experiencing loneliness, depression and stress. As for the relationship between adjustment to the university and cyberbullying, many studies have shown that suffering from bullying or cyberbullying during past academic phases (primary or secondary education) may predict a poorer university adjustment as well as psycho-social problems. Suresh and Tipandjan (2012) found that students who mentioned having been victims of cyberbullying during primary education displayed academic, interpersonal and self-esteem problems during higher education, while the university students who had suffered from cyberbullying during secondary education and those who had been victims during both earlier educational periods (primary and secondary education) presented interpersonal, family and low self-esteem problems in the university. Along these lines, Jantzer and Cashel (2017) showed that having been a victim of cyberbullying during the secondary education period may predict a poorer social and personal–emotional adjustment to the university. But there are currently few studies analyzing whether or not this adaptation to the university acts as a predictor variable to being a victim or aggressor of cyberbullying. To the best of our knowledge, the study conducted by Souza et al. (2018) is the only relevant work. These authors, using a sample of 979 Brazilian and Portuguese university students, found that newcomer adjustment and student feelings of well-being predict being a cyberbullying victim or aggressor.

This study attempts to remedy this situation by identifying variables that predict the probability of being a victim or aggressor of cyberbullying. If levels of anxiety, depression and stress or university adaptation with its distinct dimensions (academic, social, personal–emotional, and institutional), in fact predict cyberbullying, this would suggest that prevention or intervention methods should be directed specifically at these variables. So, the objective of this study is to analyze the predictive power of anxiety, depression, stress and university adaptation for being a victim or aggressor of cyberbullying in higher education. Considering the limited number of prior studies in this area, we anticipate the following: (a) high levels of anxiety, depression and stress are predictor variables of being a victim or aggressor of cyberbullying; and (b) distinct factors of university adaptation (academic adjustment, social adjustment, personal–emotional adjustment and institutional adjustment) are predictor variables of being victims or aggressors of cyberbullying.

## Materials and Methods

### Participants

Spanish university students aged 18–46 (*M* = 21.65; *SD* = 4.25) participated in the study. The sample consisted of 1282 university students (46.33% males and 53.67% females) who studied in the Early Childhood Education Master’s degree program (24.88%), Primary Education Master’s degree program (27.77%), Psychology undergraduate degree program (17.16%), Physical Activity and Sports Science undergraduate degree program (15.83%), and undergraduate degree program in Business Administration and Management (14.36%). The ethnic composition of the sample was as follows: 90.4% Spanish, 5.38% Hispanic-American, 2.97% other Europeans, 0.73% Asian, and 0.52% Arab. Based on the Chi-squared test of distribution homogeneity, it was verified that there were no significant statistic differences between the gender × course year groups (χ^2^ = 3.85; *p* = 0.312) (see Table 1).

**TABLE 1:** Distribution of the sample by sex and degree.

	**Early childhood education**	**Primary education**	**Psychology**	**Physical activity and sports science**	**Business administration and management**	**Total**
Males	92	136	76	161	129	594
Females	227	220	144	42	55	688
Total	319	356	220	203	184	1282

### Measures

#### The European Cyberbullying Intervention Project Questionnaire (ECIPQ; Del Rey et al., 2015)

The Spanish version of the European Cyberbullying Intervention Project Questionnaire (ECIPQ; Del Rey et al., 2015) was used to identify the victims and aggressors of cyberbullying in higher education. The questionnaire, consisting of 22 items, assessed two factors: Cybervictimization (11 items) and Cyberaggression (11 items) responded to using a Likert-like scale of 1–5 (1 = never; 2 = once or twice; 3 = once or twice a month; 4 = once a week; 5 = more than once a week). Students were to note to what extent they have suffered from situations of victimization or have perpetrated said situations of victimization using electronic means over the past 2 months (exclusion or dissemination of rumors, receiving or making insults, identity theft, being excluded and ignored or the manipulation of images). The questionnaire has suitable rates of internal consistency (Casas et al., 2013). In this study, the Cybervictimization and Cyberaggression subscale obtained suitable reliability indices (Cronbach’s alpha equal to 0.86 for Cybervictimization and 0.76 for Cyberaggression).

#### Depression, Anxiety Stress Scale-21 (DASS-21; Bados et al., 2005)

The Depression Anxiety Stress Scale-21 (DASS-21) is a reduced version of the Lovibond and Lovibond (1995) scale used for the assessment of depression, anxiety, and stress. Thus, the DASS-21, with a total of 21 items, considers three factors: Anxiety, Depression and Stress. The Depression subscale assessed the dysphoria, hopelessness, sadness, anhedonia, depreciation of life, self-contempt and lack of interest or involvement. The Anxiety subscale assesses aspects related to psycho-physiological activation or autonomous excitation (sweating hands, tremor, etc.), and subjective experiences of anxiety. Finally, the Stress subscale assesses the difficulty in being relaxed, nervous excitation, agitation, irritability, and impatience. This test has a satisfactory convergent validity and suitable discriminant validity (Lovibond and Lovibond, 1995; Crawford and Henry, 2003). Reliability, assessed using Cronbach’s alpha, was found to be acceptable for the three scales (Lovibond and Lovibond, 1995; Bados et al., 2005). In this study, the reliability indices were adequate, having Cronbach’s alphas of 0.87, 0.90, and 0.86 for the factors of Anxiety, Depression and Stress, respectively.

#### Student Adaptation to College Questionnaire (SACQ; Baker and Siryk, 1999)

Student adjustment was measured using the Spanish version of the SACQ (Rodríguez et al., 2012). The SACQ is a questionnaire consisting of 67 items that are responded to on a Likert-like scale of 9 points, from 1 (does not apply to me at all) to 9 (applies very strongly to me). This questionnaire assessed how the students adjusted to the university based on four dimensions: Academic Adjustment, Social Adjustment, Personal–Emotional Adjustment, and Institutional Attachment. The Academic Adjustment dimension assesses how the students faced the educational demands, considering aspects such as motivation to complete the academic requirements, academic effort and satisfaction with the academic environment (e.g., “I am up to date on the works that I am asked to complete”). The Social Adjustment dimension assesses student success with respect to interpersonal and social demands of the university environment (e.g., “In the university, I am meeting people and making friends”). The Personal–Emotional Adjustment dimension assesses the psychological state of the student and the level of general psychological anguish (e.g., “It is difficult for me to handle the stress produced by the university”). Finally, the Institutional Adjustment dimension assesses the student satisfaction with the universal experience in general, and the quality of the relationship between the student and the institution (e.g., “I am thinking about definitively quitting the university”). High scores in the distinct dimensions indicate a better student adjustment. The Spanish validation of the questionnaire received suitable internal consistency indices (Academic Adjustment α = 0.90; Social Adjustment α = 0.85; Personal–Emotional Adjustment α = 0.89; and Institutional Adjustment α = 0.85). In this study, the four subscales were found to have suitable reliability, with Cronbach values equaling 0.86 (Academic Adjustment), 0.79 (Social Adjustment), 0.86 (Personal–Emotional Adjustment), and 0.89 (Institutional Adjustment).

### Procedure

Individualized interviews were conducted with the directors of the university departments, in order to determine the study plan and to request their collaboration. The assessment instruments were administered collectively in the classrooms, highlighting the voluntary nature of the student participation and the confidentiality of the data. The mean administration time for the questionnaires was 10 min for the ECIPQ, 10 min for the DASS-21 and 15 min for the SACQ. The administration of questionnaires was carried out during the 2017–2018 academic year. The ethics committee of the University of Alicante granted the informed consent for the study to be conducted. The ethical principles of the Helsinki Declaration were considered with respect to research with human beings.

### Statistical Analyses

First, the prevalence rates were calculated for cybervictims and cyberaggressors from the total sample. To do so, victims and aggressors were considered from those obtaining scores that were higher than the mean score plus a standard deviation for the Cybervictimization and Cyberaggression factor, respectively. To examine the predictive or classificatory capacity of anxiety, depression, stress, and university adaptation on cyberbullying, a binary logistic regression analysis was conducted following the forward stepwise regression procedure based on the Wald test. The logistic model permits the estimation of the probability of an occurrence of an event or result (e.g., being a cyberbully) in the presence of one or more predictors (e.g., university adaptation). This probability is estimated using the so-called odds ratio (OR) statistic. If the OR is higher than one indicates that the increase of the independent variable leads to an increase in the probability of the occurrence of the event. On the other hand, an OR value lower than one indicates that an increase in the independent variable leads to a decrease of the probability of occurrence of the event (De Maris, 2003). The variables (victims and aggressor) were dichotomized as a function of percentiles 25 and 75, with the objective of identifying the low or high presence of the construct. The proportion of cases correctly classified by the logistic models calculated ranged between 81.4% (depression and stress) and 81.8% (personal–emotional adjustment and social adjustment) in the sample of victims, and between 80.3% (depression) and 80.5% (personal–emotional adjustment, academic adjustment, and institutional adjustment) in the sample of aggressor.

### Ethics Statement

The studies involving human participants were reviewed and approved by the Ethics Committee of the University of Alicante. The parent or legal guardians of all participants gave written informed consent in accordance with the Declaration of Helsinki (World Medical Association, 2013).

## Results

### Prevalence of Cybervictimization and Cyberaggression

Results indicate that 7% (*n* = 89) of the sample mentioned having been the victim of cyberbullying over the past 2 months, whereas 7.7% (*n* = 98) mentioned having been aggressor over the past 2 months.

### Prediction of Being a Victim or Aggressor of Cyberbullying With Respect to Anxiety, Depression, Stress and University Adaptation

**FIGURE 1:**
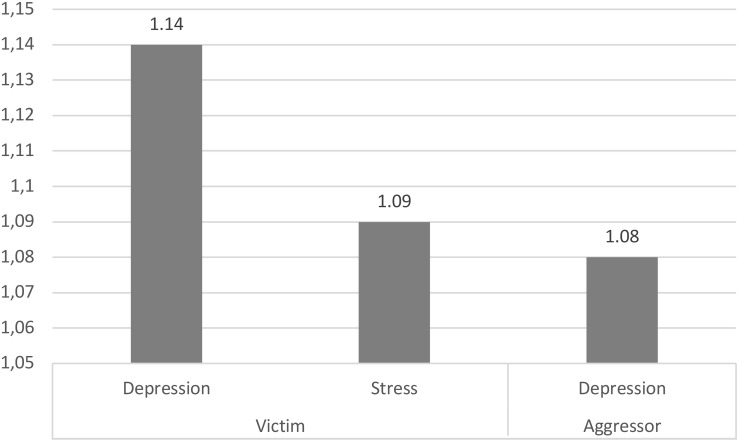
Probability of being a cyberbullying victim or aggressor based on level of anxiety, depression and stress.

Regarding the prediction of being victim or aggressor of cyberbullying with respect to anxiety, depression and stress, the OR indicate that the probability of being a victim of cyberbullying increases 14 and 9% for each point increase on the Depression and Stress scale, respectively. As for the prediction of being a cyberbullying aggressor, the OR of the logistic model indicates that students have an 8% higher probability of being a cyberbullying aggressor for each point increase in the Depression scale (see Table 2 and Figure 1).

**TABLE 2:** Logistic regression for the probability of being a cyberbullying victim or aggressor based on level of anxiety, depression and stress.

	***B***	**S.E.**	**Wald**	***p***	**OR**	**CI 95%**
**Victim**						
Depression	0.13	0.02	38.82	<0.001	1.14	1.09–1.18
Stress	0.08	0.02	12.06	<0.001	1.09	1.036–1.137
Constant	–1.94	0.11	314.86	<0.001	0.14	
**Aggressor**						
Depression	0.08	0.03	9.52	<0.001	1.08	1.03–1.139
Constant	–2.13	0.13	271.28	<0.001	0.12	

*B, coefficient; S.E., standard error; *p*, probability; OR, odds ratio; C.I., confidence interval at 95%.*

**FIGURE 2:**
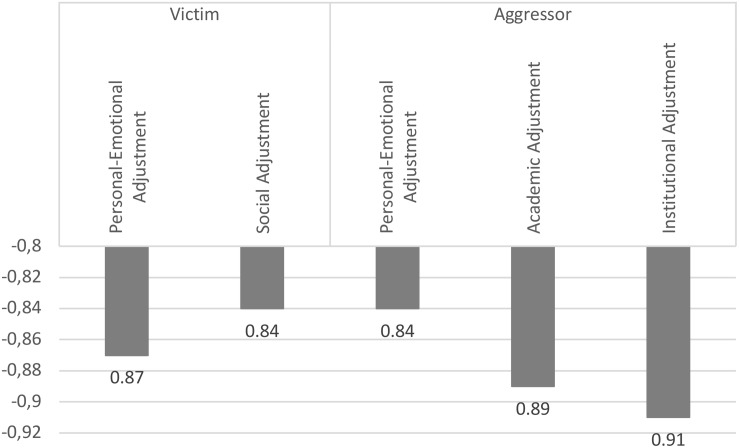
Probability of being a cyberbullying victim or aggressor based on adaptation to the university.

On the other hand, regarding to university adaptation, the OR showed that the probability of being a victim of cyberbullying decreased a 13 and 16% per unit increase in the Personal–Emotional Adjustment and Social Adjustment scale, respectively, while the probability of being an aggressor of cyberbullying decreased a 16%, 11%, and 9% for each point increase in the Personal–Emotional Adjustment, Academic Adjustment, and Institutional Adjustment scale, respectively (see Table 3 and Figure 2).

**TABLE 3:** Logistic regression for the probability of being a cyberbullying victim or aggressor based on adaptation to the university.

	***B***	**S.E.**	**Wald**	***p***	**OR**	**CI 95%**
**Victim**						
Personal–emotional adjustment	–0.02	0.03	43.95	<0.001	0.87	0.83–0.95
Social adjustment	–0.22	0.05	23.30	<0.001	0.84	0.81–0.92
Constant	0.22	0.26	0.73	<0.001	1.25	
**Aggressor**						
Personal–emotional adjustment	–0.02	0.07	5.18	<0.001	0.84	0.80–1.10
Academic adjustment	–0.03	0.04	58.26	<0.001	0.89	0.86–1.12
Institutional adjustment	0.52	0.01	23.72	<0.001	0.91	0.88–0.99
Constant	–3.80	0.61	0.39	<0.001	0.68	

**B*, coefficient; S.E., standard error; *p*, probability; OR, odds ratio; C.I., confidence interval at 95%.*

## Discussion

The goal of the present study was to verify the predictive capacity of anxiety, depression, stress and university adaptation with respect to being a victim or aggressor of cyberbullying during higher education. Cyberbullying is an ever more prevalent social problem in developed countries and has devastating consequences for all who are involved. Therefore, numerous studies have attempted to analyze this phenomenon, particularly during the period of adolescence (DeSmet et al., 2015; Ho et al., 2017). However, despite the high prevalence that has been determined (Finn, 2004; Lindsay and Krysik, 2012; Faucher et al., 2014; Zalaquett and Chatters, 2014), few studies have used the university population in their research. Higher education students, facing a significant period of changes, as well as the specific characteristics of the university cycle, present a series of elements that substantially differ from those of other students. So, this study attempts to make up for the limited number of studies considering these students, so as to obtain objective data that permits a greater knowledge of the phenomenon, in hopes of potential prevention or intervention measures.

The results of this study indicate that a percentage of university students (7%) referred to having been cyberbullying victims, with this percentage being higher in the case of aggressors, with 7.7% confessing to having been cyberbullies. These prevalence rates for both victims and aggressors coincide with that found in prior empirical studies (Finn, 2004; Zalaquett and Chatters, 2014). On the other hand, data has revealed that some emotional problematics are predictor variables of being a cyberbullying victim or aggressor. Specifically, high depression and stress rates predicts a higher probability of being a victim of cyberbullying, whereas the probability of being a cyberbully increases when students have higher depression rates, supporting hypothesis 1 of this study. Unfortunately, few studies have analyzed whether these high rates are the result of predictive variables of cyberbullying in the university setting, making the comparison of results found in this study quite complex. However, it is clear that the specific characteristics of this changing period may predict emotional problems in the overall university population (Arrieta et al., 2013; Beiter et al., 2015; Dalky and Gharaibeh, 2018). However, in this study, it is also revealed that these problematics increase the probability of being the victim or aggressor of cyberbullying, making them relevant variables when attempting to prevent or intervene in cyberbullying cases. Thus, depression predicts both the probability of being an aggressor as well as of being a victim. Depression affects the student’s social skills and self-confidence, leading to difficulties in establishing satisfactory contacts with peers, potentially leading to externalizing behaviors in aggressors. Vlachou et al. (2011) affirm that aggressive and dominating behavior in bullies come from feelings of unhappiness, dissatisfaction with life, depression and high levels of anger or rage. On the other hand, depressed individuals may experiment social isolation, a sense of meaningless, interpersonal problems and a negative self-image, making him/her more vulnerable. These difficulties and imbalances, together with other circumstances, may result in the student being more vulnerable to suffering from cyberbullying in the university setting due to this increased level of depression. Along these lines, Kaltiala-Heino et al. (2010) found that the relationship between depression and school bullying was bi-directional, such that depression acted as a predictor of bullying and bullying was a predictor of depression. Similar results were reported by Reijntjes et al. (2010), who concluded that there was indeed a bi-directional relationship between victimization and internalization problems, whereby being a victim of bullying predicted future emotional problems, while at the same time, depression, anxiety, anguish, insecurity or low self-esteem all predisposed the student to becoming a victim. Likewise, the stress produced in an educational environment is higher in the university setting, due to its greater demands (Putwain, 2007) placing these students at an emotional and social disadvantage and, thereby making them more vulnerable to cyberbullying.

On the other hand, this study has shown how certain university adjustment factors are predictive variables for being a victim or aggressor of cyberbullying, supporting hypothesis 2. Specifically, personal–emotional adjustment, and social adjustment are found to be predictor variables of being a victim of cyberbullying, with a greater personal–emotional and social adjustment decreasing the probability of being a victim. These results are coherent with previous studies that have found that student victims of cyberbullying have internalizing problems (anger/rage, discomfort, stress, worrying, fear, loneliness, helplessness, depression, shame, and indifference, etc.) (Schenk and Fremouw, 2012; Faucher et al., 2014; Giménez et al., 2015; Na et al., 2015; Aricak and Ozbay, 2016). It also supports studies that have suggested that these personal and emotional problems may lead to an increased risk of victimization (Garaigordobil and Oñederra, 2010; Reijntjes et al., 2010; Martínez-Monteagudo et al., 2019a), as well as the results of the first part of this study. As for social adjustment, many studies have suggested that victims display poor social adjustment (difficulties making friends, poor peer relationships, lack of social skills) as compared to non-victims (Kowalski et al., 2012). Therefore, the data from this study suggests that good personal–emotional and social adjustment acts as a protective factor for becoming a victim of mockery, humiliation or harassment through the digital media.

On the other hand, Personal–Emotional, Academic and Institutional Adjustment have been found to be predictor variables for being a cyberbully in the university setting, with the probability of being a bully decreasing as the levels of these adjustments increase. Although the lack of similar studies hinders a comparison of these results, the data agrees with that from prior studies that confirm the poor personal–emotional, academic and institutional adjustment of cyberbullies (Friedlander et al., 2007; Credé and Niehorster, 2012). So, with respect to personal–emotional adjustment, distinct studies have shown that having suitable personal and emotional skills is generally considered to be a protective factor from the appearance of problematic behaviors such as school bullying or cyberbullying (Garaigordobil and Oñederra, 2010; Elipe et al., 2015; Martínez-Monteagudo et al., 2019a). Emotional skills help students to feel a greater level of empathy toward their classmates, which may significantly reduce their involvement in intimidating behavior; however, one of the characteristics that is typically mentioned by cyberbullies is a low level of empathy with victims, since they do not appear to demonstrate unrest or guilt as a result of their aggressions, being unable to empathize with the victim’s emotions or feelings (Hinduja and Patchin, 2007; Aricak, 2009). On the other hand, bullies tend to display poor academic performance, with low integration in academic and scholastic dynamics (Egeberg et al., 2016). So, having a positive academic adjustment (motivation for completing academic requirements, making an effort academically and satisfaction with the academic environment) acts as a protective factor against engaging in aggressive behavior toward peers. As for Institutional Adjustment, referring to student satisfaction with the overall university experience and the quality of the relationship between the student and the institution, this is also a protective factor against becoming a cyberbully. Therefore, student satisfaction with the institutional climate of his/her university is a relevant factor to consider when attempting to prevent or intervene in cyberbullying. Universities differ, among other aspects, in their organizational structure, their co-habitation rules and in the type of relationships that are established between students and the rest of the educational community. So, while some universities have positive and integrating climates in which few students feel excluded from the teaching-learning process and feel like they belong to the institution, other universities have more negative climates in which it is more likely for situations of bullying to arise amongst students (Souza et al., 2018). Clearly, in the university adaptation process, the student’s personal characteristics play a fundamental role, but the characteristics of the institution are also clearly important.

Likewise, we believe that future studies should attempt to determine which factors could help to better understand cyberbullying. Thus, for example, the analysis of other variables, such as social support (Worsley et al., 2019), the impulsivity or other features of self-regulation (Kokkinos et al., 2014), aggressiveness (Martínez-Monteagudo et al., 2019a), sexual orientation (Wensley and Campbell, 2012), coping styles (Hu et al., 2018), emotional intelligence or family environment (Martínez-Monteagudo et al., 2019b) can help to better understand this problem. Therefore, intervention strategies should also attempt to increase the levels of these protective factors in order to decrease the levels of risk factors associated with cyberbullying.

### Limitations and Practical Implications

Finally, this study has certain limitations including its cross-sectional nature, preventing the establishment of causality. Therefore, future studies of a longitudinal nature should be carried out. Furthermore, the assessment of the variables through only self-reporting measures may lead to biases, so other methods should also be included, such as peer assessments, evaluations by professors or observational methods. Similarly, students have been classified as victims versus non-victims, and aggressors versus non-aggressors. A latent class analysis would be necessary to provide a more thorough scenario of the cyberbullying problem. Likewise, it would be interesting to analyze whether cyberbullying, emotional intelligence and adaptation to the university acts in the same way in the different university degrees, and according to the sex of the students. Finally, it should be noted that the few studies that have been carried out with university samples make results comparison difficult. Despite these limitations, this study offers valuable information for professionals, parents, students and policy and educational institutions, which may be used to create specific prevention and intervention programs in response to cyberbullying in the university setting, thereby helping to reduce the associated negative consequences. The results of this study have some major practical implications. On the one hand, it highlights the prevalence rates of cyberbullying in the university setting, bringing light to a topic that remains to be studied by science. The numerous findings in adolescents cannot be extrapolated to the university setting, due the characteristics of the latter and the changing nature of those that are involved. So, more empirical evidence is necessary to establish specific prevention and intervention programs that are adjusted to this scenario. On the other hand, the results highlight the need to identify the university students’ emotional problems, as well as their capacity to adapt to the institution, since this study shows how high depression and stress rates, in addition to having negative consequences on the student’s academic, social and emotional adjustment, also act as predictive factors of cyberbullying. So, screening systems are necessary to permit the identification of those students whose high levels of emotional alterations in order to act preventatively against cyberbullying. These psychoeducational programs emphasize procedures such as cognitive restructuring, relaxation, conflict resolution, and effective communication (Dacre and Qualter, 2012; Dalky and Gharaibeh, 2018). Also, future studies may determine why certain students who also have high levels of emotional problems are less susceptible to being victims or aggressors of cyberbullying. So, for example, the social support network, self-concept or family relationships may act as a damper for cyberbullying. So, the intervention strategies should focus on strengthening these protective factors and weakening the risk factors related to cyberbullying.

On the other hand, universities should implement educational policies that permit the suitable adjustment to the university context in order to also prevent this problem. Institutional programs should provide a safe environment of well-being for the students that permits them not only to develop suitably academically, but also personally and emotionally. The institutional prevention policies should promote active student participation against cyberbullying, establishing programs on prevention and awareness of responsible Internet use, establishing activities to develop socio-emotional and pro-social skills, promoting social awareness, the ability to resolve conflicts and coping strategies that permit them to handle distinct situations, as well as feelings of belonging and a connection to the university community.

Although cyberbullying causes major imbalances in all educational stages, perhaps in the university environment students are more unprotected (see Myers and Cowie, 2019, for a review). Cyberbullying in the university context has differentiating characteristics of the school environment that can make difficult to detect and intervene in it. The university victims of cyberbullying have reached the age of majority, beginning with their adult stage. Given this new scenario, society expects that the necessary coping resources will be available in the face of adverse situations. However, such strategies are not achieved solely because of the transition from the stage of secondary to university education. In this way, victims may not report that they are mocked, intimidated, blackmailed, etc. because they might be ashamed of not being able to solve such situations, associating this fact with a lack of maturity (Orel et al., 2017). Added to this idea, the legal implications that cyberbullying might entail are present, as they are victims and stalkers who have reached the age of majority (Myers and Cowie, 2019). Likewise, observers express less empathy and less sensitivity to the anguish of their peers in this educational stage. It is therefore necessary to improve the systems and advice to students to help them; establishing comprehensive systems in which the professionals involved are trained to deal with this problem (Myers and Cowie, 2019). Thus, researchers and specialists need to continue collaborating to design interventions and methods that allow granting useful tools for future generations to safely surf the internet.

## Data Availability Statement

The datasets generated for this study are available on request to the corresponding author.

## Ethics Statement

This study was carried out in accordance with the recommendations of Ethics Committee of the University of Alicante. The protocol was approved by the Ethics Committee of the University of Alicante. The parent or legal guardians of all participants gave written informed consent in accordance with the Declaration of Helsinki (World Medical Association, 2013).

## Author Contributions

MM-M conceived the study, participated in its design, coordination, and the statistical analyses, and drafted the manuscript. BD participated in the design of the study and data interpretation, and assisted in drafting the manuscript. JG-F assisted with the study conception and participated in the statistical analyses. All authors read and approved the final manuscript. CR-E performed a critical review of the manuscript and assisted with interpretation of the findings.

## Conflict of Interest

The authors declare that the research was conducted in the absence of any commercial or financial relationships that could be construed as a potential conflict of interest.
